# Refining visceral adipose tissue quantification: Influence of sex, age, and BMI on single slice estimation in 3D MRI of the German National Cohort

**DOI:** 10.1016/j.zemedi.2025.02.005

**Published:** 2025-03-22

**Authors:** Tobias Haueise, Fritz Schick, Norbert Stefan, Elena Grune, Marc-Nicolas von Itter, Hans-Ulrich Kauczor, Johanna Nattenmüller, Tobias Norajitra, Tobias Nonnenmacher, Susanne Rospleszcz, Klaus H. Maier-Hein, Christopher L. Schlett, Jakob B. Weiss, Beate Fischer, Karl-Heinz Jöckel, Lilian Krist, Thoralf Niendorf, Annette Peters, Anja M. Sedlmeier, Stefan N. Willich, Fabian Bamberg, Jürgen Machann

**Affiliations:** a Institute for Diabetes Research and Metabolic Diseases, Helmholtz Munich at the University of Tübingen, Tübingen, Germany; b German Center for Diabetes Research (DZD), Tübingen, Germany; c Section on Experimental Radiology, Department of Diagnostic and Interventional Radiology, University Hospital Tübingen, Tübingen, Germany; d Department of Internal Medicine, Division of Diabetology, Endocrinology and Nephrology, University of Tübingen, Tübingen, Germany; e Department of Diagnostic and Interventional Radiology, University Hospital Heidelberg, Heidelberg, Germany; f Institute of Radiology and Nuclear Medicine, Hirslanden Klinik St. Anna, Lucerne, Switzerland; g Department of Diagnostic and Interventional Radiology, Medical Center, Faculty of Medicine, University of Freiburg, Freiburg, Germany; h Division of Medical Image Computing, German Cancer Research Center, Heidelberg, Germany; i Pattern Analysis and Learning Group, Department of Radiation Oncology, University Hospital Heidelberg, Heidelberg, Germany; j Department of Epidemiology and Preventive Medicine, University of Regensburg, Germany; k Center for Translational Oncology, University Hospital Regensburg, Germany; l Bavarian Cancer Research Center (BZKF), Regensburg, Germany; m Berlin Ultrahigh Field Facility (B.U.F.F.), Max-Delbrück Center for Molecular Medicine in the Helmholtz Association (MDC), Berlin, Germany; n Experimental and Clinical Research Center, A Joint Cooperation Between the Charité Medical Faculty and the Max-Delbrück Center for Molecular Medicine in the Helmholtz Association (MDC), Berlin, Germany; o Institute for Medical Informatics, Biometry and Epidemiology (IMIBE), University Hospital Essen, Essen, Germany; p Department of Epidemiology, Institute for Medical Information Processing, Biometry and Epidemiology, Ludwig-Maximilians-Universität, Munich, Germany; q Institute of Epidemiology, Helmholtz Munich, Environmental Health Center, Neuherberg, Germany; r German Center for Cardiovascular Research (DZHK), Partner Site Munich Heart Alliance, Munich, Germany; s German Center for Diabetes Research (DZD), Partner Site Neuherberg, Neuherberg, Germany; t Institute of Social Medicine, Epidemiology and Health Economics, Charité – Universitätsmedizin Berlin, Berlin, Germany

**Keywords:** Magnetic resonance imaging, Obesity, Visceral adipose tissue, Single slice quantification, Deep learning

## Abstract

**Objectives:**

High prevalence of visceral obesity and its associated complications underscore the importance of accurately quantifying visceral adipose tissue (VAT) depots. While whole-body MRI offers comprehensive insights into adipose tissue distribution, it is resource-intensive. Alternatively, evaluation of defined single slices provides an efficient approach for estimation of total VAT volume. This study investigates the influence of sex-, age-, and BMI on VAT distribution along the craniocaudal axis and total VAT volume obtained from single slice versus volumetric assessment in 3D MRI and aims to identify age-independent locations for accurate estimation of VAT volume from single slice assessment.

**Materials and methods:**

This secondary analysis of the prospective population-based German National Cohort (NAKO) included 3D VIBE Dixon MRI from 11,191 participants (screened between May 2014 and December 2016). VAT and spine segmentations were automatically generated using fat-selective images. Standardized craniocaudal VAT profiles were generated. Axial percentage of total VAT was used for identification of reference locations for volume estimation of VAT from a single slice.

**Results:**

Data from 11,036 participants (mean age, 52 ± 11 years, 5681 men) were analyzed. Craniocaudal VAT distribution differed qualitatively between men/women and with respect to age/BMI. Age-independent single slice VAT estimates demonstrated strong correlations with reference VAT volumes. Anatomical locations for accurate VAT estimation varied with sex/BMI.

**Conclusions:**

The selection of reference locations should be different depending on BMI groups, with a preference for caudal shifts in location with increasing BMI. For women with obesity (BMI >30 kg/m^2^), the L1 level emerges as the optimal reference location.

## Introduction

The prevalence of obesity and its associated disorders concerning, for example, the endocrine or cardiovascular system, increased over the last decades [1], [2]. Therefore, precise assessment of the volume of adipose tissue (AT) compartments, as well as their regional distribution within the body, play an important role in the study of obesity, providing the potential to longitudinally monitor changes due to aging, lifestyle interventions, or medical treatment.

Quantification of visceral adipose tissue (VAT) is of particular interest, as abdominal obesity, manifested by increased VAT volume, is a key factor in determining the metabolic syndrome and the risk of type 2 diabetes and cardiovascular disease [3], [4]. Estimation of VAT using easily accessible anthropometric indices like waist circumference is limited, as this metric does not permit differentiation of VAT and abdominal subcutaneous adipose tissue (SAT) and, the latter of which is only weakly associated with an increased risk of cardiometabolic diseases [5], [6]. Despite moderate to strong correlation with imaging-based measures, anthropometric indices only explain up to 90 % of the variance of VAT and SAT when applied in linear models [7], [8], [9].

Whole-body MRI is considered the gold-standard for differentiation and assessment of SAT and VAT [10], [11], [12]. SAT and VAT can reliably be differentiated from other tissue types using standard T1-weighted images leveraging the inherent short T1 of fat [10] or applying chemical shift-encoded (CSE) techniques such as Dixon MRI [11], [12]. In conjunction with (semi-)automated segmentation-based quantification, most recently using deep learning-based approaches [11], evaluation of AT distribution and quantitative assessment of the respective compartment volumes has been applied in numerous cross-sectional and longitudinal observations focusing on MR-based phenotyping and interventional trials, e.g., for prevention of type 2 diabetes in subjects at increased risk for metabolic or cardiovascular diseases (as reviewed by Hu et al. [13]) [14], [15], [16], [17], [18]. Ongoing population-based cohort studies like German National Cohort (NAKO) [19], the UK Biobank [20] or the Framingham heart study [21] are analyzing VAT in comparison to anthropometric, metabolic and/or environmental conditions [22], [23]. Whole-body MRI used in large population imaging studies is resource intense. Routine clinical MRI examinations targeting VAT distribution assessment using Dixon MRI are typically not covering the entire trunk due to examination time constraints and the effort for AT segmentation. To reduce the examination time (and radiation exposure in CT measurements) as well as the effort for adipose tissue segmentation, single-slice abdominal MRI is often used for estimation of VAT volume using a representative slice at a defined anatomical location.

There are various landmarks which can be considered for selection of a representative slice (e.g., lumbar vertebral bodies, intervertebral disks, umbilical level). Schweitzer et al. reported the level of L3 as the best compromise site for men and women to assess total tissue volumes of VAT, by also taking SAT and skeletal muscle into account [24]. Schwenzer et al. showed strong significant correlations between total VAT volume and VAT area at the umbilical level [25] whereas Maislin et al. revealed that the level of the intervertebral disc L3/2 showed better prediction of VAT volume than L5/4 [26]. Furthermore, it has been shown that the amount of VAT and its distribution along the craniocaudal axis is different for women and men [27]. Body composition also depends on age and BMI. In most studies, the influence of BMI and age on VAT distribution along the craniocaudal axis is neglected.

Aim of this study was to use fat-selective Dixon MRI of the trunk and automatically generated segmentations of visceral adipose tissue (VAT) from >11,000 participants in a large, population-based cohort study to 1) systematically analyze the accumulation and distribution of VAT along the craniocaudal axis with respect to sex, age and BMI and 2) to investigate the impact of sex-, age-, and BMI-related differences on single-slice MRI assessment of VAT, i.e., on selection of a reference location for single-slice image acquisition best reflecting VAT volume.

## Materials and methods

### German National Cohort

The German National Cohort (NAKO, *NAKO Gesundheitsstudie)* is a population-based, multicentric prospective cohort study in Germany enrolling >200,000 participants selected randomly from the general population. Its main objective is to identify and characterize risk factors for major chronic diseases (e.g. type-2 diabetes and cancer) [28]. For a subset of approximately 30,000 participants, whole-body MRI examinations have been conducted at five imaging sites applying standardized neurologic, cardiovascular, thoracoabdominal, and musculoskeletal imaging protocols [19]. All local institutional review boards in charge of the five imaging sites approved the NAKO (i.e., all study documents including protocols, participant information documents, and declaration of consent forms). Written informed consent of all participants was obtained before study enrollment.

### Data acquisition

Standardized thoracoabdominal MRI was performed at five sites using 3 T whole-body scanners (MAGNETOM Skyra, Siemens Healthcare, Erlangen, Germany). MRI of the body trunk was performed in supine position using a dedicated T1-weighted 3D VIBE two-point Dixon technique in axial orientation (80 partitions per slab, partition thickness 3 mm, in-plane voxel size 1.4 × 1.4 mm, echo times 1.23 and 2.46 ms, repetition time 4.36 ms, flip angle 9°, bandwidth 975 Hz/Pixel, acquisition time 12 s). A spine-array and two body-array RF coils (Siemens Healthcare, Erlangen, Germany) were used for signal reception [19]. Fat- and water-selective images were automatically calculated using the MR vendor’s image reconstruction pipeline. The data used in this study were obtained from the first NAKO release of MRI data, which includes 11,191 participants being screened between May 2014 and December 2016.

Body height and weight were assessed using standardized measuring instruments across the study centers (Stadiometer 274 for height and medical Body Composition Analyzer 515 for weight, seca GmBH, Hamburg, Germany) [29].

### Sample selection

The study sample comprised 11,191 individuals from NAKO with available MR data. This cohort has been previously reported [11]. This prior article dealt with the feasibility of deep learning-based automatic assessment of adipose tissue compartments (i.e., quantification and spatial distribution) in a large population-based cohort whereas in this study we report on the influence of sex, age, and BMI on the spatial distribution of VAT along the craniocaudal axis and the consequences of sex-, age-, and BMI-related differences on systematic errors using single-slice estimation of total VAT.

**Figure 1:**
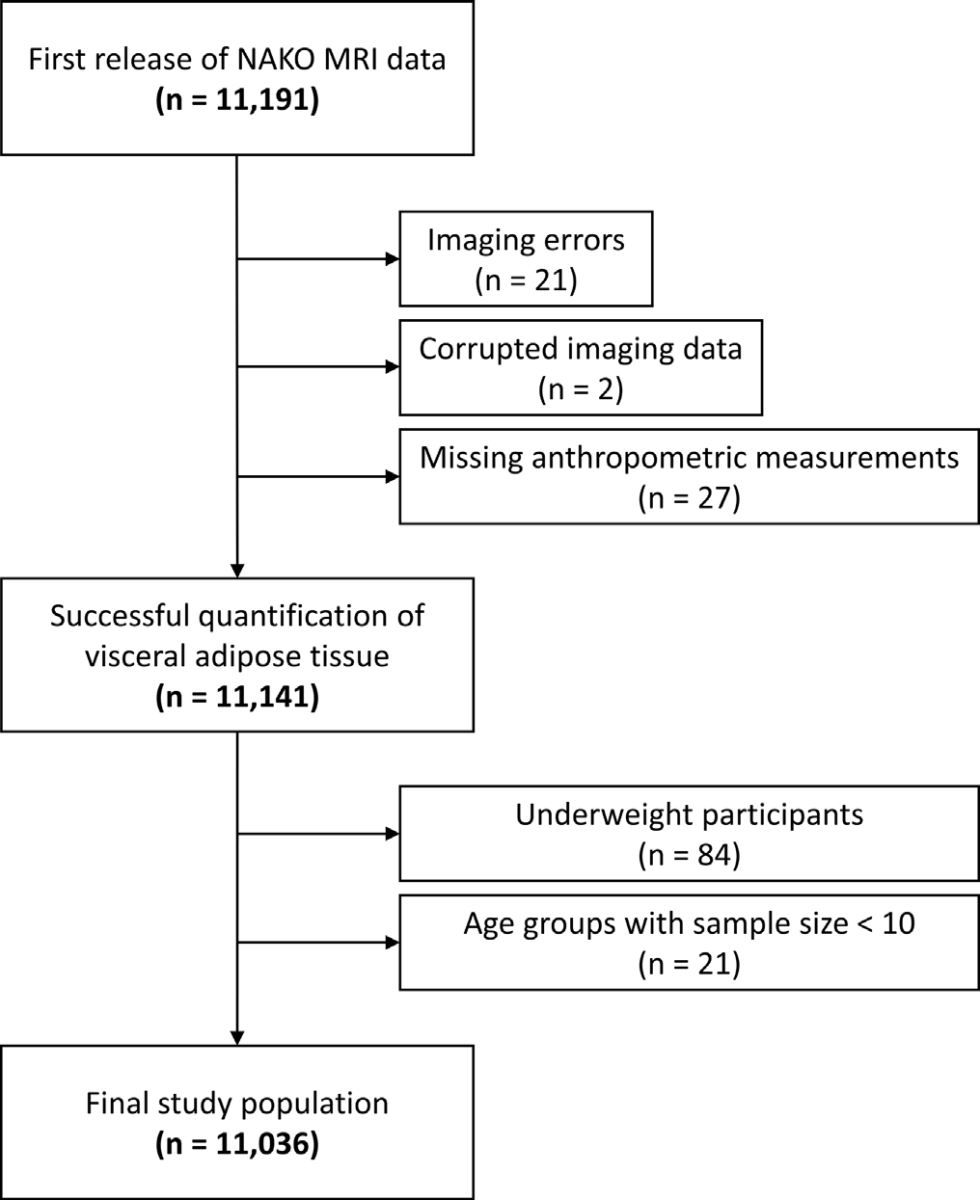
**Flowchart of the study.** Application of exclusion criteria to the first release of NAKO Dixon MRI data.

Aim of the analysis was to provide descriptive values, not effect estimates. Therefore, a formal power calculation was not conducted in this study. Participants were excluded if MR data contained severe artifacts, e.g., partial fat-water swaps, or were corrupted. Incomplete anthropometric measurements, e.g., missing data for height or weight, led to exclusion. For BMI- and age-related analyses, the participants were categorized into five BMI groups (I: 18.5–24.9 kg/m^2^, II: 25–29.9 kg/m^2^, III: 30–34.9 kg/m^2^, IV: 35–39.9 kg/m^2^, V: >40 kg/m^2^) and five 10-year age groups (i: 20–29 years, ii: 30–39 years, iii: 40–49 years, iv: 50–59 years, v: >60 years). Underweight participants (BMI <18.5 kg/m^2^) were excluded (n = 84) due to small total number of participants resulting in very small age groups. Due to the small total number of individuals older than 70 years (n = 135), these were subsumed with the previous category (i.e., >60 years). BMI-dependent age groups with sample size n <10 were excluded. A flowchart of the study is presented in Fig. 1.

### Derivation of craniocaudal VAT profiles

**Figure 2:**
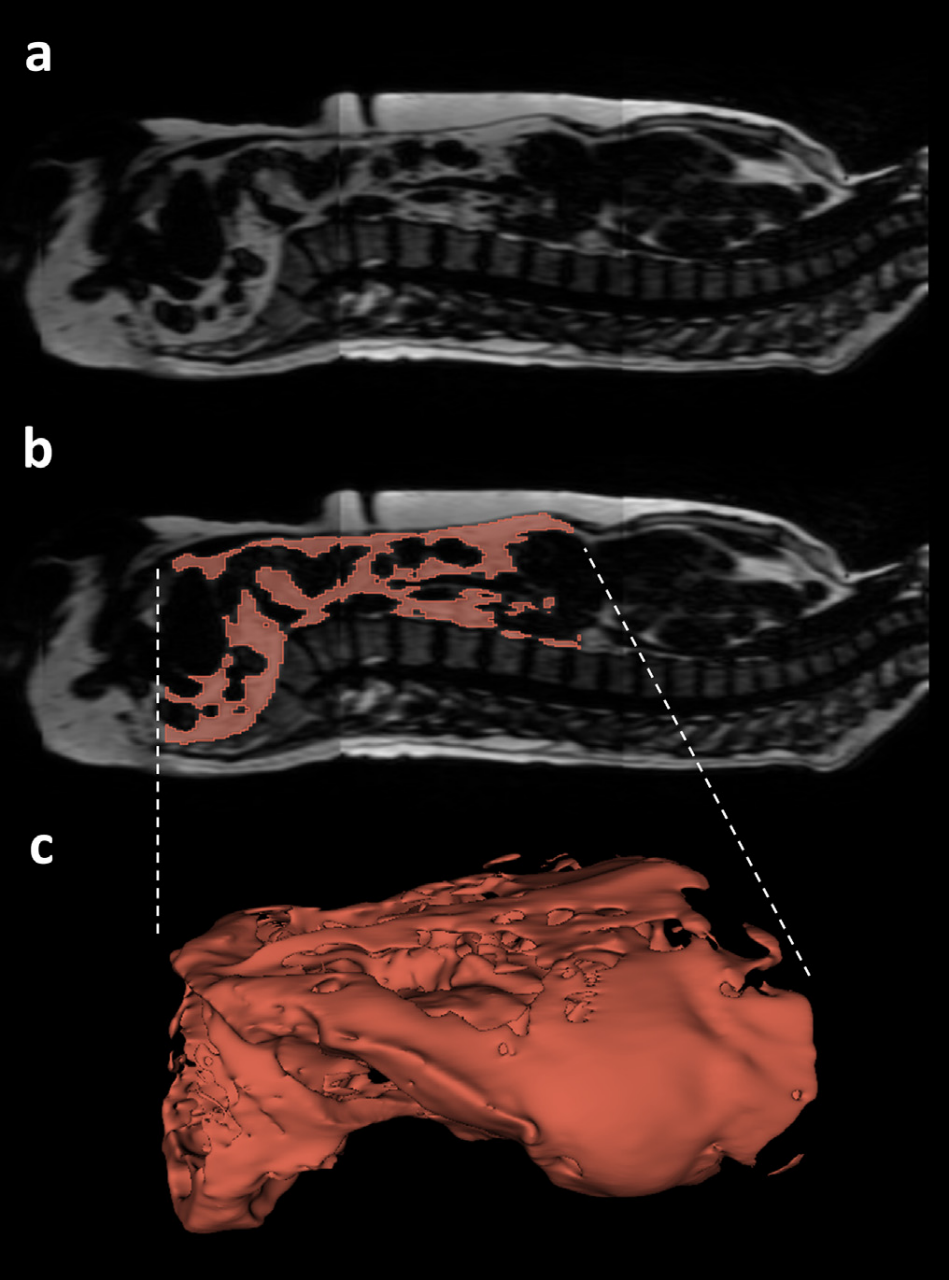
**Example of visceral adipose tissue segmentation.** a) Fat-selective sagittal image of a 50-year old man (BMI 24.6 kg/m^2^) including the spine for anatomical reference b) Corresponding segmentation of visceral adipose tissue (VAT) c) Three-dimensional representation of VAT segmentation.

**Figure 3:**
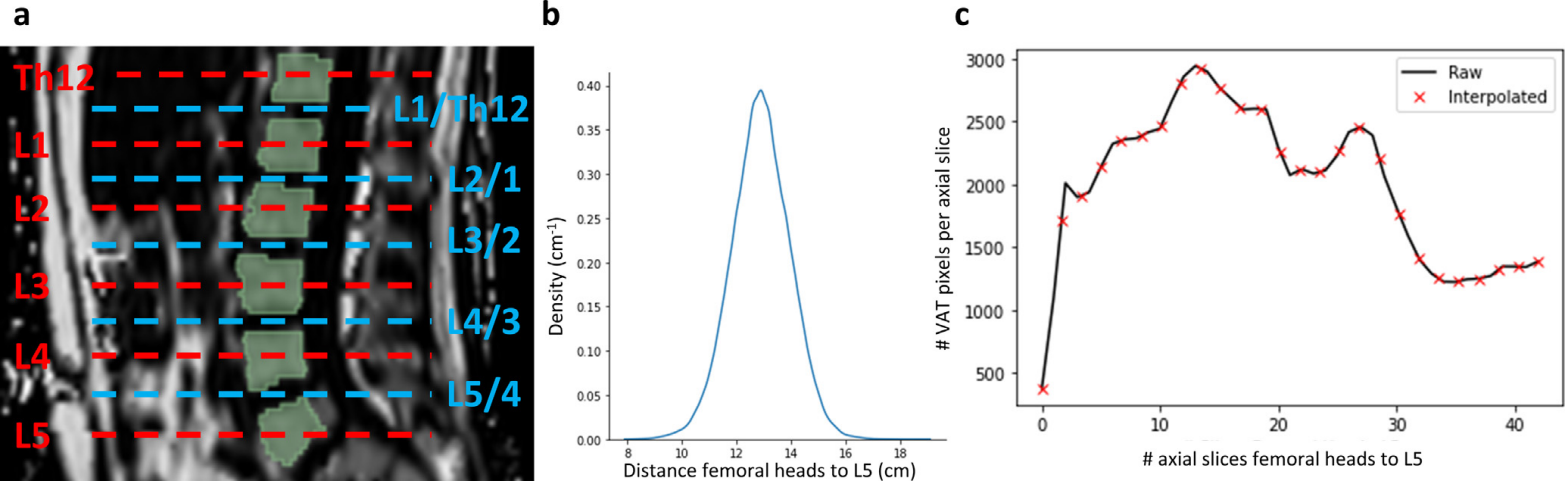
**Standardization of visceral adipose tissue to body locations.** a) Anatomical locations of AT evaluations derived from automatic segmentation of the spine. b) Distribution of distance between femoral heads and lumbar vertebra L5 in the complete study population (12.9 ± 1.1 cm). c) Exemplary interpolation of axial VAT profile to obtain a standardized step size of 0.5 cm.

Whole-body VAT masks and respective VAT volumes were obtained from fat-selective MR images using a previously published and publicly available deep learning-based automatic U-Net segmentation model (https://zenodo.org/records/7229667) [11] covering the trunk from femoral heads to the cardiac apex [10] in a standardized manner. Dice coefficient of VAT segmentation based on five-fold cross-validation was indicated with 0.947 ± 0.033. Relative and absolute errors were reported as −1.25 ± 2.79 % and −18.4 ± 51.1 ml, respectively. Fig. 2 shows an example of the segmentation of VAT of a 50-year old man (BMI 24.6 kg/m^2^) including the spine for anatomical reference. The three-dimensional representation (Fig. 2c) illustrates differences in VAT expansion in the pelvic and abdominal cavities. Craniocaudal VAT profiles were derived from segmented masks by considering the per-slice percentage of total VAT in axial images. By combination with computer-generated spine segmentations [30], VAT profiles were matched and standardized to distinctive anatomical locations, namely the center of eight vertebral bodies (L5 to Th10) and seven corresponding intervertebral discs (see Fig. 3a), thereby eliminating direct influences of height of the individuals. To standardize and identify locations of VAT located caudally, i.e., between L5 and the femoral heads, a scale was generated by the following steps: 1) measuring the mean distance from the center of L5 to the center of the femoral heads (derived from standardized VAT segmentations) using the MRI slice thickness of 3 mm (see Fig. 3b), 2) linear interpolation of the number of VAT pixels in axial images with a fixed number of equidistant data points (see Fig. 3c), and 3) assessment of cross-sectional VAT area at these specified locations. In this study, the mean distance of 12.9 ± 1.1 cm and linear interpolation of the number of VAT pixels in axial images with 26 data points to generated a scale with a resulting step size of 0.5 cm.

### Age-independent single-slice VAT estimation

Within each BMI group per sex, age-stratified intersections, i.e., anatomical location with the smallest difference of VAT percentage between all age groups, in craniocaudal VAT profiles were assessed to achieve independence of age. Identified locations of intersection were selected for single-slice estimation of VAT volume: Measured axial VAT cross-sectional area from a single slice was multiplied by a sex- and BMI-specific scaling factor (reciprocal of single-slice VAT percentage) from the cohort data.

$${\mathrm{VAT}}_{\mathrm{total}/estimated}\left[l\right]\approx {\mathrm{VAT}}_{\mathrm{single}-slice/measured}\left[cm^{2}\right]∙\frac{{\mathrm{VAT}}_{\mathrm{total}/Sex|BMI}}{{\mathrm{VAT}}_{\mathrm{single}-slice/Sex|BMI}}\left[1000\;cm^{2}\right]$$

### Statistical analysis

Data are presented as mean ± standard deviation unless stated otherwise. For the assessment of the accuracy of the 2D assessment, the association of single-slice VAT estimates with 3D reference volumes was assessed using linear regression and Pearson’s correlation coefficient. Gaussian kernel density estimation was used to estimate the distribution of measured distances from the center of the femoral heads to the center of lumber vertebra L5. *p* <0.01 was considered statistically significant. Statistical analyses were performed using R 4.3.2 (https://www.r-project.org/).

## Results

Due to the exclusion criteria, n = 155 participants were removed (n = 21 due to fat-water swaps in Dixon imaging, n = 2 due to corrupted image data, n = 27 due to missing anthropometric measurements, n = 84 due to BMI <18.5 kg/m^2^, and n = 21 due to subgroup sample size less than 10 participants; see Fig. 1); the final study sample consisted of n = 11,036 participants (mean age, 52 ± 11 years, 5681 men, see Table 1). Almost half (48 %) of the men were in BMI group II (25–29.9 kg/m^2^), whereas 48 % of the women were categorized into BMI group I (18.5–24.9 kg/m^2^). There was no significant difference in age between women (mean age 51.8 ± 11.3 years) and men (mean age 52.1 ± 11.4 years); t(11034) = 1.33, *p*  =  0.18. Difference in BMI was significant between women (mean BMI 26.3 ± 5.1 kg/m^2^) and men (mean BMI 27.4 ± 4.1 kg/m^2^); t(11034) = 12.39, *p* < 0.001. For all BMI groups, men had on average about 73 % more VAT compared to women. More than 95 % of the included participants self-identified as White individuals.

**Table 1:** **Study population.** Overview and anthropometric measures in men and women. BMI in five groups (I: 18.5–24.9 kg/m^2^, II: 25–29.9 kg/m^2^, III: 30–34.9 kg/m^2^, IV: 35–39.9 kg/m^2^, V: >40 kg/m^2^). Data are presented as mean ± standard deviation with range in parentheses.

**BMI Group**	**I**	**II**	**III**	**IV**	**V**	**Total**
**Men**						
n	1690	2733	979	234	45	5681
Age (years)	48.4 ± 12.3	53.3 ± 10.8	54.4 ± 10.3	55.4 ± 9.2	54.0 ± 8.2	52.1 ± 11.4
	(20–72)	(22–72)	(20–72)	(32–71)	(41–67)	(20–72)
Height (cm)	179.0 ± 6.9	178.1 ± 6.9	177.6 ± 7.0	176.9 ± 7.1	175.3 ± 8.3	178.2 ± 7.0
	(156.5–204.2)	(155.3–204.6)	(154.5–200.2)	(152.8–192.6)	(162.8–197.0)	(152.8–204.6)
Weight (kg)	74.1 ± 7.2	86.6 ± 7.8	101.1 ± 8.6	115.7 ± 10.5	130.8 ± 16.2	86.9 ± 14.0
	(48.6–97.3)	(64.7–117.8)	(79.5–129.6)	(83.6–144.0)	(110.9–191.6)	(48.6–191.6)
BMI (kg/m^2^)	23.1 ± 1.4	27.3 ± 1.4	32.0 ± 1.4	36.9 ± 1.3	42.4 ± 2.3	27.4 ± 4.1
	(18.5–24.9)	(25.0–29.9)	(30.0–34.9)	(35.0–39.9)	(40.0–49.4)	(18.6–49.4)
VAT (l)	2.8 ± 1.3	5.0 ± 1.7	7.0 ± 1.8	8.7 ± 2.1	9.7 ± 2.2	4.8 ± 2.4
	(0.5–9.4)	(0.6–13.8)	(1.6–14.2)	(4.0–15.3)	(5.1–14.7)	(0.5–15.3)
**Women**						
n	2577	1676	743	260	99	5355
Age (years)	49.4 ± 11.7	53.5 ± 10.7	55.0 ± 10.2	56.0 ± 9.6	54.7 ± 7.3	51.8 ± 11.3
	(20–72)	(20–72)	(21–72)	(30–72)	(40–72)	(20–72)
Height (cm)	166.0 ± 6.5	164.4 ± 6.5	163.4 ± 6.3	163.1 ± 6.4	162.3 ± 6.8	164.9 ± 6.5
	(144.5–189.0)	(141.4–188.7)	(144.5–188.4)	(148.1–183.7)	(126.4–179.3)	(126.4–189.0)
Weight (kg)	61.4 ± 6.4	73.5 ± 6.8	85.8 ± 7.4	98.6 ± 8.5	115.6 ± 11.8	71.4 ± 14.0
	(40.1–86.7)	(53.5–97.4)	(64.9–117.7)	(77.7–121.5)	(75.8–157.5)	(40.1–157.5)
BMI (kg/m^2^)	22.3 ± 1.6	27.2 ± 1.4	32.1 ± 1.4	37.0 ± 1.4	43.8 ± 3.2	26.3 ± 5.1
	(18.5–24.9)	(25.0–29.9)	(30.0–34.9)	(35.0–39.9)	(40.0–53.9)	(18.5–53.9)
VAT (l)	1.5 ± 0.8	2.8 ± 1.1	4.1 ± 1.3	5.1 ± 1.3	5.8 ± 1.4	2.5 ± 1.5
	(0.2–5.9)	(0.5–7.9)	(1.2–9.7)	(1.8–8.6)	(2.8–10.3)	(0.2–10.3)

VAT: visceral adipose tissue.

**Figure 4:**
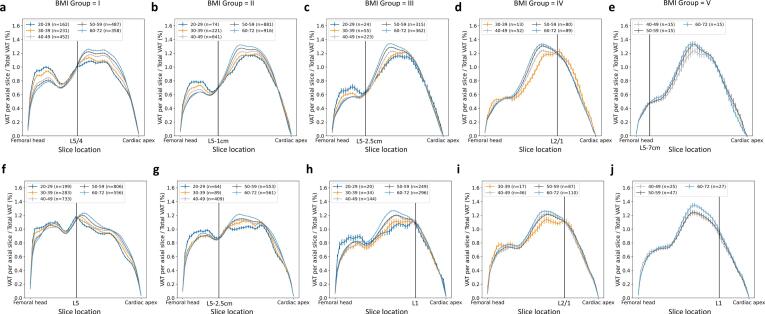
**Craniocaudal profiles of visceral adipose tissue.** Automatically quantified visceral adipose tissue (VAT) per slice in relation to total VAT (percentage share in %) per BMI group and different age groups in men (a–e) and women (f–j) (BMI Groups I: 18.5–24.9 kg/m^2^, II: 25–29.9 kg/m^2^, III: 30–34.9 kg/m^2^, IV: 35–39.9 kg/m^2^, V: >40 kg/m^2^). Intersections of age-independence are marked by a vertical line. Error bars represent standard error of the mean.

Visually, craniocaudal VAT profiles showed sex-, BMI- and age-related differences in VAT distribution along the body axis (see Fig. 4a–e for men and Fig. 4f–j for women). For women and men, two distinct areas/plateaus of VAT accumulation were identified, separated by a boundary approximately 3 cm below L5 presumably corresponding to the pelvic inlet separating pelvic and abdominal cavities. These regions became more differentiated as BMI increased, with the caudal (pelvic) level decreasing and the abdominal peak increasing. In women, the caudal (pelvic) level of VAT accumulation was generally higher compared to men. Within each BMI group, younger age groups exhibited more pelvic VAT. This trend reversed in older age groups, where older individuals had more VAT per axial slice in the abdomen. This effect was most pronounced in men of BMI Group I (see Fig. 4a), whereas age differences became less pronounced with increasing BMI. In women, age-dependent differences were particularly visible in the abdominal region across BMI groups I to IV (see Fig. 4f–i). Additionally, there was a steeper curve in abdominal VAT accumulation with increasing BMI. The shape of the VAT profile curves became more similar between women and men as VAT increased. In men, regardless of the BMI group, the largest percentage of VAT was located at the L3 level. In women, the level of maximum VAT percentage shifted from L5/4 to L3 with increasing BMI.

**Figure 5:**
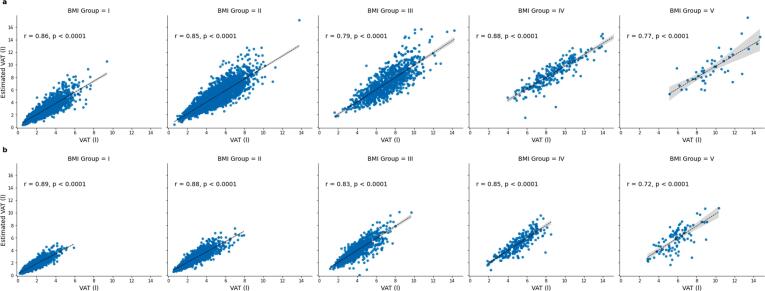
**Correlation analysis.** BMI-dependent correlations of estimated VAT volume and VAT volume in men (a) and women (b) (BMI Groups I: 18.5–24.9 kg/m^2^, II: 25–29.9 kg/m^2^, III: 30–34.9 kg/m^2^, IV: 35–39.9 kg/m^2^, V: >40 kg/m^2^).

Anatomical locations of intersection for age-independent single-slice VAT volume estimation differed between women and men and varied with respect to BMI (see Table 2). Notably, intersections are shifted cranially for women with BMI >30 kg/m^2^. Derived age-independent single-slice VAT volume estimates showed good correlation with VAT volume in both sexes (Pearson’s r ranging 0.77–0.88 in men and 0.72–0.89 in women, respectively, see Fig. 5).

**Table 2:** **Locations of age-independent single slice VAT estimation.** BMI-dependent and sex-specific factors for VAT volume estimation from single-slice VAT area (BMI Groups I: 18.5–24.9 kg/m^2^, II: 25–29.9 kg/m^2^, III: 30–34.9 kg/m^2^, IV: 35–39.9 kg/m^2^, V: >40 kg/m^2^).

**BMI Group**		**I**	**II**	**III**	**IV**	**V**
Men	Location Factor	L5/4 103	L5-1 cm 147	L5-2.5 cm 173	L2/1 84	L5-7 cm 195
Women	Location Factor	L5 88	L5-2.5 cm 118	L1 97	L2/1 91	L1 104

**Figure 6:**
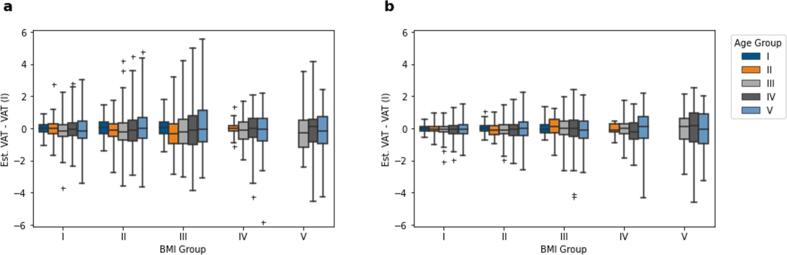
**Error analysis.** BMI- and age-dependent VAT estimation difference in men (a) and women (b). Statistical outliers in the boxplots are marked with ‘+’.

Fig. 6 shows BMI- and age-dependent errors for the estimation of VAT volume using the described methods of age-independent factors compared to volumetric quantification. The range of the errors is increased with age, whereas mean errors over the complete study population were –0.07 ± 0.99 L in men and −0.06 ± 0.56 L in women, corresponding to relative mean absolute errors of 1.44 % and 2.37 %, respectively.

## Discussion

Visceral adiposity is recognized as a primary driver of cardiometabolic risk [3]. Imaging-based methods are the gold standard for measuring specific adipose tissue and skeletal muscle depots [9]. This study investigated the spatial distribution of visceral adipose tissue (VAT) along the craniocaudal axis and examined sex-, age-, and BMI-related effects on single-slice measurement. For women and men, axial percentage of total VAT volume at standardized anatomical landmarks differs depending on age and BMI yielding different scaling factors. Elimination of age effects using the point of intersection in craniocaudal VAT profiles provides the possibility of a reliable direct derivation of total VAT volume from slice-wise VAT percentage.

As reviewed by Hu et al. [13] and reinforced by more recent studies [31], [32], [33], the selection of representative slices for single-slice assessment of VAT is still subject of ongoing debates. Particularly in analyses based on diagnostic CT examinations and time constrained clinical practice, efficient and reliable single-slice estimation of VAT volume could be a preferred measure. Schweitzer et al. proposed that a single-slice image at L3 was the best trade-off to assess VAT in men and women [24]. Additionally, Schweitzer et al. investigated the influence of age indicating that independent of age (mean age of 38 years compared to 68 years), VAT volume is best estimated at L3 [34]. With regard to BMI-related effects (mean BMI of 29.5 kg/m^2^), the findings of Schaudinn et al. are in line with previous results by using intervertebral disk L3/2 as a reference [31] using CT. However, the influence of age and BMI was seldom considered together due to limitations in sample size.

Although the MR methodology (Dixon-based imaging) as well as automated assessment, e.g., AI-based segmentation, of VAT are well established, our approach is dedicatedly focused on the distribution of VAT along the body axis – with individual and standardized co-registration of high-resolved images to the spine – and thereby bringing the volumetric information back to a recommendation where to analyze single-slice measurements for a proper estimation of total VAT volume. This has been done considering the influence of sex, age, and BMI and is therefore in contrast to previous analyses just giving correlations between single-slice and volumetric data ignoring these influencing factors. Furthermore, our findings regarding craniocaudal VAT distribution were based on a rather large cohort and can also be transferred to other imaging modalities such as CT, where adipose tissue can be reliably quantified by defined Hounsfield units showing excellent correlation to MR-derived VAT volume [35], [36]. In addition, the results are not limited to the chemical-shift-based MR contrast applied in the NAKO protocol, but can be transferred to MRI techniques with other contrasts, where fat can distinctly be differentiated from lean tissue, as T1-weighted (fat is brighter than other tissues due to shorter longitudinal relaxation time) or T2-weighted MRI (fat is brighter than most other tissues due to longer transverse relaxation time).

However, single-slice quantifications are still only an approximation and cannot capture all the information contained in a volumetric assessment. Own unpublished data revealed, that VAT volume has a higher correlation with insulin sensitivity compared to a single-slice approach considering the umbilical level. Whether this holds true in our study population and how this translates to, e.g., cardiometabolic risk estimation needs to be investigated further.

This study has limitations. First, standardization of anatomical landmarks based on automatic segmentation of the spine and linear interpolation is a potential source of bias. Second, standardized landmarks located caudally to L5 may be limited in practical applicability. Third, the application of BMI thresholds leads to ambiguities of our results, especially when considering women with a BMI around 30 kg/m^2^: From our cross-sectional data, we cannot derive a clear recommendation as to whether an individual woman should be considered as belonging to BMI group II, and therefore should ideally be measured using a single slice at 2.5 cm below L5, or to BMI group III. However, as the estimation of total VAT from single-slice measurements is always approximate, the error should be neglectable. Forth, our analysis is based on the perspective of variation in metabolically active VAT in relation to sex, age, and BMI and the direct influence on the selection of a single slice location. Thereby, we neglect other adipose tissue compartments, for example, the role of subcutaneous adipose tissue (SAT) or the VAT-over-SAT ratio, and possible technical optimizations, for example, multi-slice estimation. Combination of slices will improve the accuracy of total VAT estimation [37]; however, this analysis is focused on the edge case of single-slice measurements. Further, our analyses and recommendations of taking sex and BMI into account for selection of a representative single-slice location are limited to a cohort randomly drawn from compulsory registries of residents and, thus, comprises a rather inhomogeneous (but not necessarily representative) selection of the general population in Germany. Additionally, over 95% of the participants in the NAKO self-identified as White individuals, and thus, the presented results most likely reflect only this group with generalizable accuracy. Furthermore, we have not yet investigated, whether the distribution of VAT differs in patients with metabolic diseases (e.g., type-2 diabetes), congestive or chronical heart failure, hypothyroidism, lipedema and/or carcinosis, all of them leading to weight gain and therefore accumulation of VAT. This needs to be evaluated in further studies. From our cross-sectional results it remains unanswerable, whether single-slice observations can be used to reliably quantify changes of VAT after lifestyle intervention.

## Conclusion

In a large cohort study, a comprehensive assessment of volumetric body fat distribution and its predictability from single-slice estimation showed that besides sex, also additional age- and BMI-dependent factors need to be considered in the selection of a reference location: VAT volume estimation using single-slice measurements should be shifted caudally starting from L5 with increasing BMI in men and women, but for women with obesity (BMI >30 kg/m^2^) L1 should be preferred.

## Funding

This work was funded by the Deutsche Forschungsgemeinschaft (DFG, German Research Foundation) – 428224476/SPP2177; and in part by a grant from the Federal Ministry of Education and Research (BMBF) to the German Center for Diabetes Research (DZD e.V.) [01GI0925]. This project was conducted with data (Application No. NAKO-308) from the German National Cohort (NAKO) (www.nako.de). The NAKO is funded by the Federal Ministry of Education and Research (BMBF) [project funding reference numbers: 01ER1301A/B/C, 01ER1511D, 01ER1801A/B/C/D and 01ER2301A/B/C], federal states of Germany and the Helmholtz Association, the participating universities and the institutes of the Leibniz Association.

## Acknowledgements

We thank all participants who took part in the NAKO study and the staff of this research initiative.

## CRediT authorship contribution statement

**Tobias Haueise:** Writing – original draft, Validation, Software, Methodology, Investigation, Formal analysis, Data curation, Conceptualization. **Fritz Schick:** Writing – review & editing, Supervision, Project administration, Funding acquisition. **Norbert Stefan:** Writing – review & editing, Project administration. **Elena Grune:** Writing – review & editing. **Marc-Nicolas von Itter:** Writing – review & editing. **Hans-Ulrich Kauczor:** Writing – review & editing, Funding acquisition, Conceptualization. **Johanna Nattenmüller:** Writing – review & editing, Project administration, Funding acquisition, Conceptualization. **Tobias Norajitra:** Writing – original draft, Validation, Software, Methodology, Investigation, Formal analysis, Data curation, Conceptualization. **Tobias Nonnenmacher:** Writing – original draft, Validation, Software, Methodology, Investigation, Formal analysis, Data curation, Conceptualization. **Susanne Rospleszcz:** Writing – review & editing, Supervision, Funding acquisition, Conceptualization. **Klaus H. Maier-Hein:** Writing – review & editing, Software, Funding acquisition. **Christopher L. Schlett:** Writing – review & editing, Project administration, Funding acquisition, Conceptualization. **Jakob B. Weiss:** Writing – review & editing. **Beate Fischer:** Writing – review & editing. **Karl-Heinz Jöckel:** Writing – review & editing, Conceptualization. **Lilian Krist:** Writing – review & editing. **Thoralf Niendorf:** Writing – review & editing. **Annette Peters:** Writing – review & editing. **Anja M. Sedlmeier:** Writing – review & editing. **Stefan N. Willich:** Writing – review & editing. **Fabian Bamberg:** Writing – review & editing, Supervision, Resources, Project administration, Methodology, Funding acquisition, Conceptualization. **Jürgen Machann:** Writing – review & editing, Supervision, Project administration, Methodology, Funding acquisition, Conceptualization.

## Declaration of competing interest

The authors declare the following financial interests/personal relationships which may be considered as potential competing interests: Tobias Haueise reports financial support was provided by Deutsche Forschungsgemeinschaft. If there are other authors, they declare that they have no known competing financial interests or personal relationships that could have appeared to influence the work reported in this paper.
